# Duality of 5-HT Effects on Crayfish Motoneurons

**DOI:** 10.3389/fphys.2019.01280

**Published:** 2019-10-22

**Authors:** Julien Bacqué-Cazenave, Pascal Fossat, Fadi A. Issa, Donald H. Edwards, Jean Paul Delbecque, Daniel Cattaert

**Affiliations:** ^1^ University of Bordeaux, CNRS, Institut de Neurosciences Cognitives et Intégratives d’Aquitaine (INCIA) UMR5287, Bordeaux, France; ^2^Neuroscience Institute, College of Arts and Sciences, Georgia State University, Atlanta, GA, United States

**Keywords:** serotonin, motoneuron, excitatory and inhibitory balance, crayfish (*Procambarus clarkii*), locomotor network, sensori-motor interactions, serotoninergic neuron

## Abstract

Serotonin (5-HT) is a major neuromodulator acting on the nervous system. Its various effects have been studied in vertebrates, as well as in arthropods, from the cellular and subcellular compartments up to the behavioral level, which includes the control of mood, aggression, locomotion, and anxiety. The diversity of responses of neurons to 5-HT has been related to its mode of application, the diversity of 5-HT-receptors, and the animals’ social status history. In the locomotor network of socially isolated crayfish, the duality of 5-HT-evoked responses (excitatory/inhibitory) on motoneurons (MNs), sensorimotor pathways, and their consequences on motor network activity has largely been studied. The aim of the present report is to examine if this duality of exogenous 5-HT-evoked responses in the crayfish locomotor network can be reproduced by direct activation of 5-HT neurons in the case of socially isolated animals. Our previous studies have focused on the mechanisms supporting these opposite effects on MNs, pointing out spatial segregation of 5-HT receptors responsible either for positive or negative responses. Here, we report new findings indicating that excitatory and inhibitory effects can be achieved simultaneously in different leg MNs by the activation of a single 5-HT cell in the first abdominal ganglion.

## Introduction

The neuromodulator serotonin (5-HT) has widespread effects in the physiology of animals and humans, particularly controlling changes in behavior and motor activity in rat (Cazalets et al., 1992), insects (Buhl et al., 2008), and crustacean stomatogastric ganglion (Zhang and Harris-Warrick, 1994). In crustaceans, serotonin is particularly known to trigger dominant-like posture (Livingstone et al., 1980; Kravitz, 1988), to influence aggressiveness and the decision to retreat (Huber et al., 1997a,b; Huber and Delago, 1998; Bacqué-Cazenave et al., 2018), to modulate social status (Huber et al., 1997b, 2001), and to control escape behavior (Teshiba et al., 2001), as well as anxiety-like behavior (Fossat et al., 2014, 2015; Bacqué-Cazenave et al., 2017). Some transient effects of 5-HT, as observed during anxiety-like behavior, appear to involve brain circuits because 5-HT levels are increased in brain tissues but not in ventral nerve cord (Fossat et al., 2014), while other more durable effects of 5-HT involve changes in the ventral nerve cord (Yeh et al., 1996; Cattaert et al., 2010).

In the ventral nerve cord of crayfish, the walking leg postural network consists of sensory-motor circuits controlling leg joints, and is well studied for the second joint responsible for upward and downward leg movements (El Manira et al., 1991; Le Bon-Jego and Cattaert, 2002). At this joint, a proprioceptor, the coxo-basal chordotonal organ (CBCO), mediates a resistance reflex *via* monosynaptic connections (El Manira et al., 1991) and polysynaptic pathways (Le Bon-Jego et al., 2004), and so plays an important role in the control of leg’s posture. *In vitro,* the application of 5-HT on the walking leg postural network evokes several cooperative effects involving changes in membrane potential, input resistance (*R*_in_), and time constants of motoneurons (MNs), and in the level of activation of polysynaptic excitatory pathways of the resistance reflex (Le Bon-Jego et al., 2004). However, the amplitude of these effects is very variable among the MN pools in a single experiment (Le Bon-Jego et al., 2004). Moreover, the social status of the animal determines the sign of the response to 5-HT, being excitatory in dominant and inhibitory in subordinate animals (Cattaert et al., 2010). In a first attempt to decipher the origin of excitatory and inhibitory responses evoked by 5-HT, we previously used local micro-application of 5-HT on the walking leg postural network and showed that two types of responses could be evoked in the same depressor MN depending on the location of the puff electrode on the neuron (Bacqué-Cazenave et al., 2013). When 5-HT was applied close to the initial segment of MNs, the response of Dep MNs was inhibitory (opening of a presumed K^+^ channel, leading to hyperpolarization of the membrane and drop in input resistance), while a more central application in the neuropile resulted in an excitatory response that involved closing of a presumed K^+^ channel resulting in membrane depolarization and increase of input resistance.

However, very little is known about the natural source of 5-HT that could induce such modulatory effects. In the present report, we have studied the effect of one source of 5-HT [a pair of 5-HT cells in the first abdominal (A1) ganglia] on the walking leg postural network of crayfish. Indeed 5-HT immunocytochemical studies have demonstrated the presence of pairs of large, anteriorly projecting 5-HT cells in the fifth thoracic (T5) and first abdominal ganglia (A1) (Beltz and Kravitz, 1983, 1987; Beltz, 1999). It is therefore possible that the response to 5-HT reflects the sensitivity of the walking leg postural network to 5-HT released by these cells. However, it is not known if these 5-HT cells project onto the postural circuit, or whether the activation of the 5-HT cells could elicit the several cooperative effects observed with 5-HT application (Le Bon-Jego et al., 2004) and whether these effects would be excitatory or inhibitory.

In the present study, we addressed these questions using an *in vitro* preparation of the crayfish fifth walking leg postural network (El Manira et al., 1991) that permitted intracellular recordings to be made of both leg depressor motoneurons from the fifth thoracic ganglion (T5) and the left 5-HT cell from the first abdominal ganglion (A1). In order to eliminate the variations of responses to 5-HT due to social status, experiments were performed on socially isolated crayfish (Yeh et al., 1996). Our results show that (1) the activation of a single 5-HT cell can induce mixed excitatory and inhibitory effects on walking leg MNs; (2) these effects induce functional changes in the walking leg postural network by modifying the amplitude of the response of MNs to mechanosensory inputs; (3) the induced effects are multiple and cooperative, and they involve modifications of the intrinsic properties (input resistance, membrane potential) of Dep MNs; and (4) the effects on intrinsic properties of Dep MNs are direct, while the effects on sensory-motor response involve polysynaptic pathways.

## Materials and Methods

### Experimental Animals

Experiments were performed on male adult crayfish (*Procambarus clarkii*) weighing 25–30 g. The animals were collected locally and maintained while individually isolated during 3 weeks before experiments. They were kept at 18–20°C on a 12:12 h light:dark cycle, and fed once a week with shrimp pellets and carrots.

### *In vitro* Preparation

**Figure 1:**
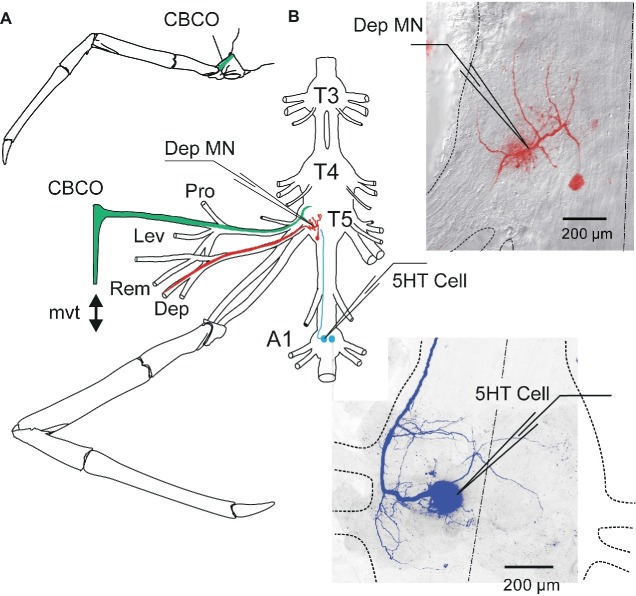
*In vitro* preparation of crayfish walking leg postural system of fifth thoracic leg and first abdominal 5-HT cell. **(A)** Location of the coxo-basipodite chordotonal organ (CBCO) in the fifth walking leg. **(B)** The *in vitro* preparation of the crayfish thoracic locomotor system consists of thoracic ganglia 3–5 (*T3*, *T4*, *T5*) and the first abdominal ganglion (*A1*) dissected out together with motor nerves of the proximal muscles (promotor, *Pro n*; remotor, *Rem n*; levator, *Lev n*; depressor, *Dep n*) and the *CBCO*, a proprioceptor that encodes the vertical movements of the leg. A mechanical puller allowed us to mimic the vertical movements (*mvt*) of the leg by stretching and releasing the CBCO strand. The central nervous system was isolated from the CBCO by a Vaseline wall in order to superfuse only the ganglia with low calcium ringer. Intracellular recordings from depressor motoneurons (Dep MN) were performed within the neuropile of the fifth thoracic ganglion and from the left 5-HT cell of the first abdominal ganglion with glass microelectrodes. Right panels: disposition of the microelectrodes used for intracellular recording from Dep MN (in the main neurite) and 5-HT cell (in the cell body), respectively.

An *in vitro* preparation of the thoracic nervous system with motor nerves innervating the coxa-basis joint and the coxo-basipodite chordotonal organ (CBCO) of the fifth leg (Figure 1A) was used (Sillar and Skorupski, 1986; El Manira et al., 1991). Prior to dissection, each animal was chilled in iced-water for 30 min. Then it was decapitated and the thorax and abdomen were pinned dorsal side-up. A section of the ventral nerve cord containing the last three thoracic (*T3–T5*) and the first (*A1*) and second (*A2*) abdominal ganglia was dissected out with all the motor nerves of the two proximal segments of the left fifth leg: promotor (Pro), remotor (Rem), anterior levator (Lev), and depressor (Dep) (Figure 1B). The oxo-basipodite chordotonal organ (CBCO), which monitors the movements of the second joint (coxo-basipodite – Figure 1A), was also dissected out and kept intact. The distal end of its elastic strand was attached to an electromagnetic puller VT101 (Ling Dynamic Systems, Meudon-la-Forêt, France) controlled by a home-made function generator that allowed the application of sinewave movements to the CBCO strand (Figure 1B) to mimic upward (during stretch) and downward (during release) movements of the leg.

The preparation was pinned dorsal side up on a Sylgard-lined Petri dish (Dow Corning Corp., Wiesbaden, Germany). The nervous system was continuously superfused with oxygenated control saline composed of (in mM) 195 NaCl, 5 KCl, 13 CaCl_2_, 2 MgCl_2_, and 3 HEPES (Sigma Chemical, St Louis, MO) with a pH of 7.65. In some experiments, a high-divalent cation solution containing (in mM) 34 CaCl_2_ and 6.4 MgCl_2_, with the sodium concentration reduced accordingly to preserve the osmolarity of the solution, was used to raise the spiking threshold of the interneurons. The fourth and fifth thoracic ganglia and the first abdominal ganglion were desheathed to improve the superfusion of the central neurons and to allow for intracellular recordings from depressor MNs (Figure 1B, red inset) and ipsilateral 5-HT cell (Figure 1B, blue inset). For sensory-motor networks to be in a quiet state (no rhythmic activity), preparations were left resting for 1 h before starting experiments.

### Sensory-Motor Circuit Studied

The depressor motoneurons (MNs) receive sensory input from the CBCO. This organ consists of an elastic strand of connective tissue that is attached proximally to the dorsal edge of the coxopodite and distally to the base of an apodeme at the proximal-dorsal edge of the basipodite (Figure 1A). Embedded within this strand are about 40 sensory neurons that project to the ipsilateral neuropil and make mono- and polysynaptic connections with the depressor MNs (El Manira et al., 1991; Le Bon-Jego and Cattaert, 2002). Half of the CBCO sensory neurons are activated when the CBCO strand is stretched, while the other half are activated when the band is released (El Manira et al., 1991). Thus, this proprioceptive organ monitors movements of the limb in the vertical plane. In the *in vitro* preparation, a pin holds the proximal end of the CBCO while a mechanical puller imposes movements to its distal end (Figure 1B).

The responses of depressor MNs to releasing movements of the elastic strand of the CBCO (that would therefore be involved in resistance reflex) were recorded intracellularly with a microelectrode generally for one MN and extracellularly for the others *via* a wire electrode on the corresponding motor nerve. To ensure that the CBCO was not damaged during the dissection, we recorded from the CBCO nerve and only used preparations with robust sensory neuron activity in response to imposed movements of the CBCO strand. In order not to damage the CBCO during the experiment, stretch movements were applied starting from the most released position of the CBCO strand, and the total amplitude of the movement was one-third of the released CBCO strand length (1–1.8 mm). The imposed CBCO movement was monitored on an oscilloscope (voltage trace) and stored on computer.

### Recordings and Electrical Stimulation

Extracellular recordings from the motor nerves innervating the depressor and levator muscles and from the sensory nerve of the CBCO were made using stainless steel pin electrodes contacting the nerves and insulated with Vaseline. The differential extracellular signals were amplified 2,000–10,000 times and filtered (high-pass 30 Hz, low-pass 3 KHz, 50-Hz notch filter) using Grass Instruments AC amplifiers (model P511J). The bath solution was grounded using a small silver plate that was chlorided using chlorine bleach. Stimulation of nerves was done with a programmable pulse generator (Master-8, A.M.P.I.) and a stimulus isolation unit (A.M.P.I.). Intracellular recordings from depressor MNs (Dep MNs, Figure 1B) and 5-HT cell (5-HT Cell, Figure 1B) were performed with glass micropipettes (Clark Electromedical Instruments, Reading, UK) filled with 3 M KCl (resistance 10–20 MΩ). The intracellular electrodes were connected to an Axoclamp 2B amplifier (Axon Instruments Inc., Foster City, CA) used in current-clamp mode. Since long recording with such electrodes could lead to changes in neuron properties (Hooper et al., 2015), we verified that no modifications in Dep MN properties occurred over time in the absence of 5-HT cell stimulation. Moreover, intracellular recordings in the same cell were maintained during less than 1 h. In addition, 5-HT cell effects on MN were observed at the beginning of stimulation. In crustacea, the somata of MNs lie outside of the neuropile (the region in which neurons form their synaptic contacts; see Figure 1B) and are linked to the arbor of the neuron by a thin passive neurite, and so contribute marginally in the electrical activity of the neuron. For these reasons, intracellular recordings were made from the main neurite where excitatory post-synaptic potentials (EPSPs) could be recorded (see Figure 1B, top right). To measure input resistance, 10 short (generally 500 ms) negative current pulses (−1 nA) were injected in the recording cell. The balance of electrode was verified and rectified if necessary. Then, the traces were averaged and the difference of membrane potential before and during pulses (at the steady state) was measured.

Depressor MNs were identified following the procedure used in Hill and Cattaert (Hill and Cattaert, 2008). Briefly, electrical stimulation of the Dep nerve would produce a spike in the Dep MN whose activity is being recorded intracellularly, and injection of current pulses in the Dep MN would produce spikes recorded in the Dep nerve. The resting membrane potential of MNs was usually in the range of −80 to −65 mV. Stability of resting membrane potential during the experiment was used as a criterion for evaluation of cell health during recordings. In crustacean MNs, soma and neurites do not actively convey spikes. Therefore, spike amplitude was generally small (<20 mV) at the recording site.

### Anatomical and Immunohistochemical Techniques

**Figure 2:**
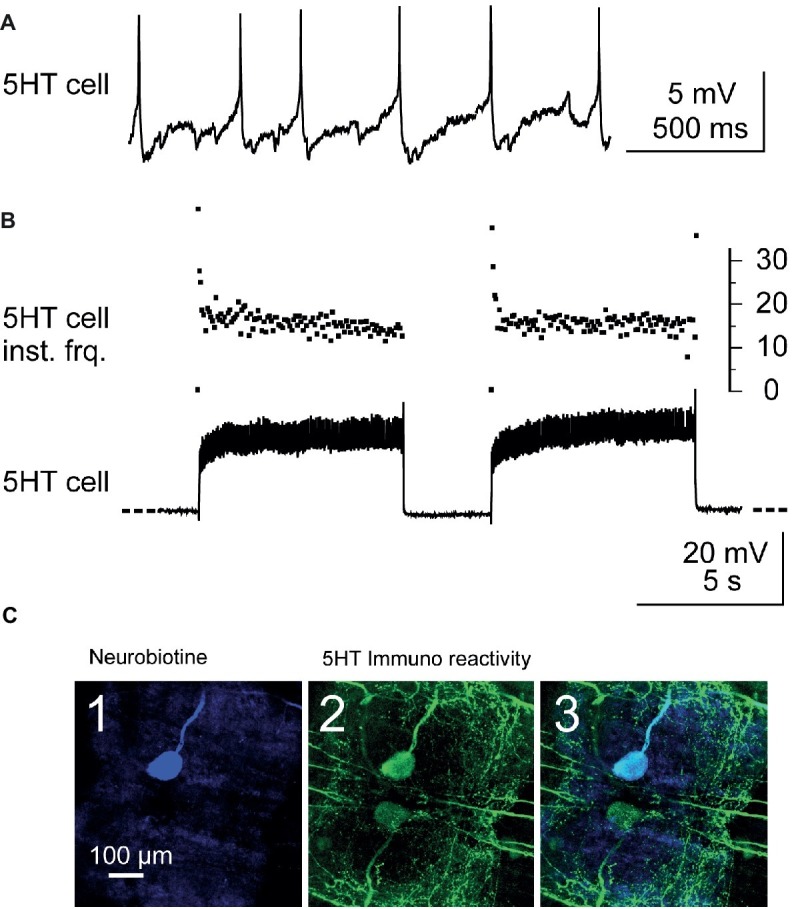
Identification and stimulation of 5-HT cells. **(A)** Intracellular recording from a A1 5-HT cell. **(B)** Stimulation of a 5-HT cell by injection of depolarizing current pulses into the cell body (lower trace). Note the fast adaptation of the instantaneous discharge frequency (*5-HT cell inst. Frq*.) at the beginning of each current pulse (upper trace): the two first spikes may fire at up to 40 Hz and after the following two spikes, the discharge frequency decreases and stabilizes around 17 Hz. **(C)** Immunostaining against 5-HT was performed after each experiment in order to confirm that the stimulated neuron was the 5-HT cell. The stimulated neuron was injected with neurobiotin and revealed with Cy5 (ABC kit) and observed in confocal microscopy. The cell body of this neuron appears in blue when illuminated in dark red light **(C1)**. At the same location, a cell body is immunoreactive to 5-HT (green, **C2**). A superimposition of the two images confirms that the injected neuron is a 5-HT cell **(C3)**.

In the first experiments, 5-HT-cells were injected with 5% dextran tetramethylrhodamine (3,000 molecular weight, Molecular Probes) in 0.2 M potassium acetate, with the electrode shank filled with 2 M potassium acetate. Neurons were injected for 1 h using square-wave pulses (500 ms duration at 1 Hz). Good results were obtained by injecting +10 nA for 5% dextran tetramethylrhodamine. However, in most experiments, neurobiotin was used instead of dextran rhodamine because it is a smaller molecule that allows injection of a larger current in the 5-HT cell without blocking the electrode. In this case, neurobiotin was revealed with an ABC kit (Avidin: Biotinylated enzyme Complex from Vector laboratories) coupled to a fluorescent dye (Cy5) (see Figure 2C).

Ganglia were fixed overnight at 4°C in 4% paraformaldehyde in a 0.2 M phosphate buffer solution (pH 7.4) and rinsed for 4 h six times in 50 ml of 0.2 M phosphate buffer solution. Preparations were then incubated for 36 h at 4°C in a ^1^/_2500_ dilution of anti-serotonin antibody (Sigma-Aldrich.) generated in rabbits against a formaldehyde cross-linked serotonin-bovine serum albumin (BSA) conjugate. Following primary antibody treatment, tissues were rinsed 4 h in phosphate buffer solution with 0.5% triton X-100 and then post-incubated for 36 h at 4°C with a secondary antibody which was goat anti-rabbit IgG labeled with fluorescein isothiocyanate (FITC) diluted 1:40. Tissues were finally rinsed in 0.2 M phosphate buffer for 6 h. Ganglia were then dehydrated in series of ethanol solutions of ascending strength (50, 70, 90%, 10 min each; 95, 100%, 2 times, 10 min each), cleared in methyl salicylate (Sigma-Aldrich), and mounted in Eukitt (O.Kindler, Germany).

The ganglia were then imaged using a confocal microscope (BX51 Olympus Fluoview 500) and the resulting digital images were analyzed using Fluoview software (Olympus).

### Data Acquisition and Analysis

Data were digitized and stored onto a computer hard disk through an appropriate interface (Power1401) and software (Spike2) from Cambridge Electronic Design Ltd. (Cambridge, UK).

Data were analyzed using the Spike2 analysis software. Spikes recorded from the CBCO nerve were identified according to their waveform based on a template matching protocol (wavemark). Templates were built automatically and corresponded to the mean duration of sensory spikes (about 1.5 ms in duration). The sampling rate for CBCO nerve recording was set to 15 kHz, which resulted in templates containing 20–22 points. The procedure used two criteria to identify a spike: (1) more than 90% of the points should be in the confidence limits of the template and (2) the maximum amplitude change for a match was less than 5%. This procedure was applied off-line. After the completion of this protocol, each identified CBCO unit (spike shape) was assigned an arbitrary number. Subsequently, a spike triggered average was performed for each CBCO unit, allowing us to observe in a given MN the occurrence of any postsynaptic events related to this unit.

### Identification of 5-HT Neurons

**Figure 3:**
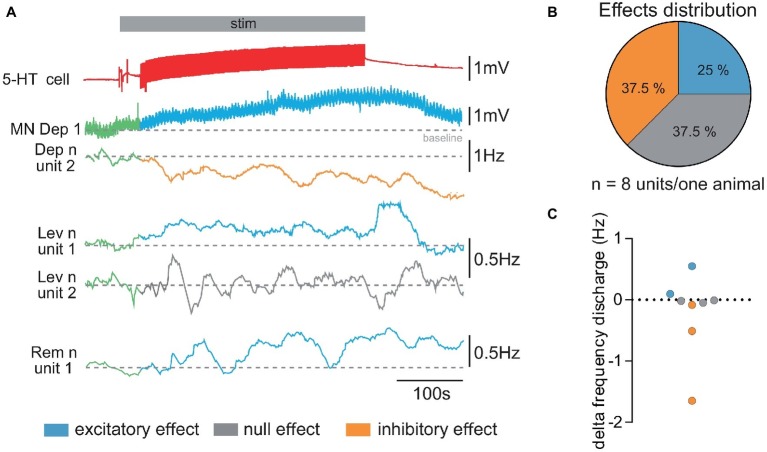
Diversity of responses from walking leg MNs of the fifth left thoracic ganglion to stimulation of the ipsi 5-HT cell. **(A)** Diversity of responses obtained in a single experiment. The 5-HT cell stimulation (*stim* in gray horizontal bar) elicits a depolarizing response in the intracellularly recorded depressor MN (*MN Dep1*). Simultaneously, the activity of several other MNs (mean firing frequency, 100 s) was extracted from extracellular recording of the Dep, Lev, and Rem motor nerves to illustrate the diversity of responses elicited by 5-HT cell stimulation: the activity of unit 2 (*Dep n unit2*) decreases (orange trace); the anterior levator nerve (*Lev n*) unit 1 presents an increased discharge (blue trace), while unit 2 does not display any change in response (gray trace) to 5-HT cell stimulation. One unit of the remotor nerve presents an increased activity (blue traces). Note that in order to analyze changes in the sensory motor response, CBCO movements were continuously applied. This resulted in “wavelets” in the intracellular recording of the Dep1 MN. **(B)** From the same experiment, the distribution of excitatory, inhibitory, and no effects was calculated from the changes in mean firing frequency measured for each recorded motor unit. **(C)** Variety of the changes in firing frequency induced by activation of the 5-HT cell (5-HT response amplitude) in motor units recorded in Pro, Rem, Lev, and Dep motor nerves from the same experiment. Discharge frequency was calculated over 200 s before activation of 5-HT cell (control, in green, in **A**) and after the end of the stimulation (decrease in orange, increase in blue, and no effect in gray in **A**).

In this study, A1 5-HTcells on the same side as recorded Dep MNs (i.e., left side) were intracellularly stimulated. The 5-HT cells were intracellularly recorded from their cell body on the ventral side of the nerve cord (the nerve cord was turned ventral side up between T5 and A1). The 5-HT cells were identified from their morphology, the location of their cell body in the anterior medial region of A1, and their electrophysiological properties (their resting membrane potential was in the range −35 to −45 mV, their discharge frequency was about 3–5 Hz – Figure 2A). Their discharge frequency reached 15–25 Hz during injection of depolarizing current pulses (40 pulses, 7 s duration, 0.1 Hz – Figure 2B). Injection of continuous current was used in some experiments (see Figure 3B). However, in most experiments, it was very difficult to get continuous firing rate of 20 Hz, and therefore, current pulses were used.

Before starting the recording, the 5-HT cell was prevented from firing by injection of hyperpolarizing current for 10 min. After the experiment, the recorded 5-HT cell was injected with neurobiotin (Figure 2C) (see methods) using depolarizing current pulses, and after fixing the preparation, immunohistochemistry against 5-HT was used (see below) to assess that the recorded neuron was a 5-HT cell (Figure 2C3). Injection of pulses of depolarizing current was controlled with a programmable pulse generator (Master-8, A.M.P.I.) connected to the current generator of the axoclamp2B. On the 36 experiments performed in this study to stimulate the left 5-HT cell, immunostaining confirmed only 16 neurons as 5-HT cells. Data on 5-HT cells presented in this paper are from these 16 experiments.

### Statistical Analyses

Statistical analyses were done with the Prism program v7 (GraphPad Software, San Diego, CA). A normal distribution was verified (D’agostino and Pearson normality test), and the results were expressed as the means ± SEM. To assess the significance of 5-HT cell activation effects on each of the parameters (*V*_m_, *R*_in_, sensorimotor response amplitude) over all experiments, mean values were calculated for each MN before and after 5-HT cell activation. Then a paired *t*-test was used to assess significant difference between the averaged responses recorded before and after 5-HT cell stimulation for each MN. Previously, we demonstrated that 5-HT had direct significant excitatory or inhibitory effect on Dep MNs (Bacqué-Cazenave et al., 2013), and since excitatory and inhibitory effects observed after 5-HT cell stimulation were analyzed separately, we used one-tailed paired *t*-test. For statistical analyses, *n* represents the number of MN recording for each parameter tested and *N* the number of experiments (animals) from which the values were extracted. In some cases, we recorded two different depressor MNs from the same animal. The effects of 5-HT cell on MN response to CBCO-imposed movements were assessed by measuring the amplitude of the response to each movement cycle. Twenty cycles were measured in control and during 5-HTcell activation. The effects of 5-HT cell on EPSP recording from MNs were assessed by measuring and averaging EPSP amplitude at peak and 15 ms after peak in the late part of the decay phase.

## Results

The unilateral projections of the A1 5-HT neurons into the fifth thoracic ganglion (Beltz and Kravitz, 1983; Spitzer et al., 2005) and the putative anatomical connectivity between them and the depressor neurons (Bacqué-Cazenave et al., 2013) suggested that the 5-HT neurons were likely to modulate the activity of the depressor neurons. To address this hypothesis, we stimulated the left 5-HT cell of the first abdominal ganglion, while extracellularly recording the activity of the motor nerves and intracellularly recording from depressor MNs from the fifth left thoracic ganglion.

### Diversity of Effects of 5-HT-Cell Activity on Leg Motoneurons’ Activity

Because our previous studies showed that local micro-applications of 5-HT could modulate the activity of MNs either excitatory or inhibitory depending on the location, we first tested if 5-HT cell stimulation could produce these two effects on the spontaneous activity of leg MNs. Stimulating the first abdominal 5-HT cell generally resulted in a global activity change of the fifth thoracic ganglion walking leg postural network (Figure 3) i.e., the motor activity recorded from each of the proximal leg motor nerves, promotor (Pro), remotor (Rem), anterior levator (Lev), and depressor (Dep) presented a global change of their firing frequency (from 7.15 ± 1.76 Hz in control condition to 6.86 ± 1.78 Hz after 5-HT cell activation; paired *t*-test, *p* = 0.20, *N* = 7). However, when we analyzed the discharge of each motor unit, we observed that some MNs increased their firing frequency, while other MNs were inhibited. Figure 3A presents such an experiment, in which the intracellularly recorded depressor MN (*MN Dep1*) is depolarized, while the extracellular recording of another MN in the depressor nerve (*Dep n unit2*) shows a decrease of its firing frequency. The other motor nerves of the fifth left leg also display a variety of responses. For example, in the anterior levator nerve (*Lev n*), unit 1 shows an increased mean discharge, while unit 2 does not display a significant change of the mean discharge in response to 5-HT cell stimulation. In the remotor motor nerve, unit 1 presents a visible activation by 5-HT cell stimulation. In this experiment, the effect of 5-HT cell increased the activity of 25% of the identified MNs in the two proximal motor nerves, decreased the activity of 37.5% of these MNs, and left 37.5% of MNs unchanged (Figure 3B). It is important to note that in the seven experiments in which the effect of 5-HT cell activation on motor unit activity was analyzed, we always found excitatory and inhibitory effects. Over the 27 motor units identified in these seven experiments, 10 MNs displayed an excitatory response, 12 MNs presented an inhibitory response, and five MNs did not change their activity (see Figure 7A). Note that most MNs were silent in such experiments, and therefore their response to 5-HT was not visible if no intracellular recording was performed. In this experiment as in others, the level of activity of the walking leg postural network was very low (below 5 Hz for all motor units), which explains why the changes in frequency were also small (below 0.5 Hz, Figure 3C).

**Figure 4:**
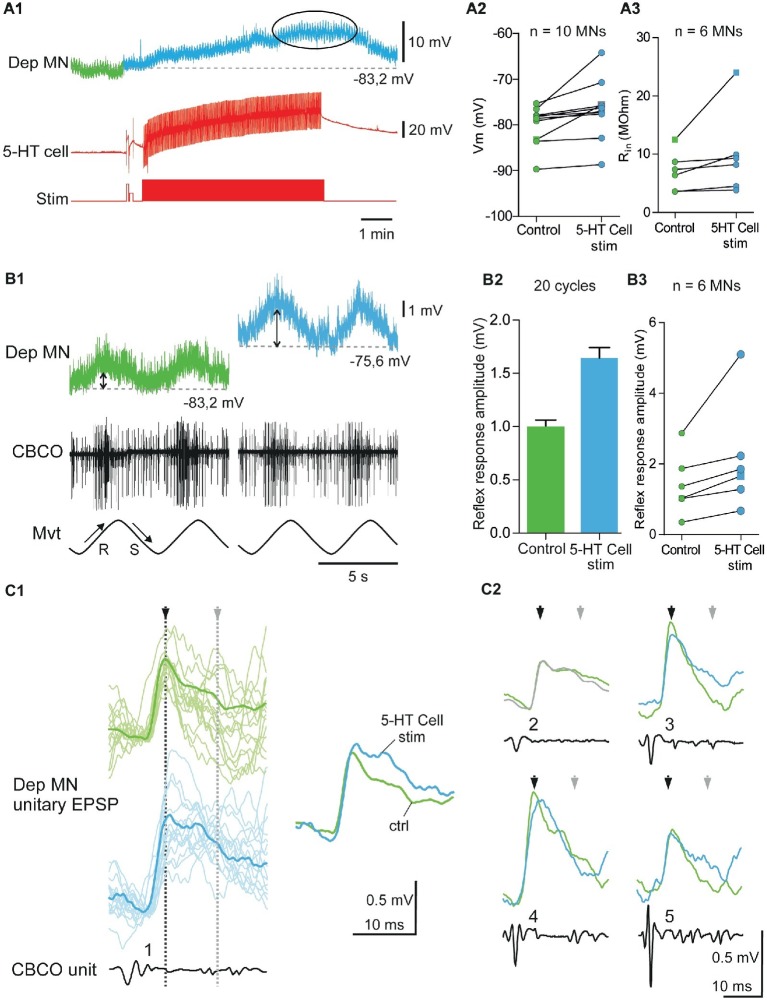
Excitatory effects induced by 5-HT cell activation on Dep MNs. **(A1)** Depolarizing response of an intracellularly recorded Dep MN to the ipsilaterally projecting A1 5-HT cell intracellular stimulation. **(A2)** Excitatory effect of 5-HT cell activation on 10 Dep MNs. Square symbol represents trace used in **A1**. **(A3)** Increased input resistance (*R*_in_) from six MNs when 5-HT cells are stimulated. Membrane potential of these MNs increases also. Square symbol represents trace used in **A1**. **(B1)** Increase of the amplitude of the sensory-motor response of an intracellularly recorded Dep MN during 5-HT cell activation. In this experiment (same as panel **A1**), sinewave movements (*Mvt*) were continuously applied to the CBCO strand and evoked sensory discharges recorded from the CBCO sensory nerve (*CBCO*). Before 5-HT cell stimulation (left column), the Dep MN depolarized during CBCO release movements (corresponding the leg levation in intact animal) and repolarized during CBCO stretch. After 5 min of 5-HT cell stimulation (right), the Dep MN potential depolarized from −83.2 to −75.6 mV and the amplitude of the sensory-motor response increased. **(B2)** In this experiment, the 5-HT cell stimulation significantly increased the amplitude of the Dep MN sensory-motor response from 0.99 ± 0.06 mV (averaged from 20 cycles) in control condition to 1.64 ± 0.099 mV (averaged from 20 cycles) after 5-HT cell stimulation. **(B3)** Increase of sensory-motor response recorded from six MNs, after the 5-HT cell stimulation. **(C1)** Examples of unitary EPSPs’ shape changes induced in Dep MN that showed a depolarizing response to 5-HT cell activation. In control condition and after 5-HT cell stimulation, superimposed examples (raw data, light traces) and corresponding average EPSP traces (dark traces) are presented. **(C2)**: Mean EPSP amplitudes measured at peak (black arrows) and 15 ms after peak in the late part of the decay phase (gray arrows). These unitary EPSPs are recorded in the same Dep MN.

**Figure 5:**
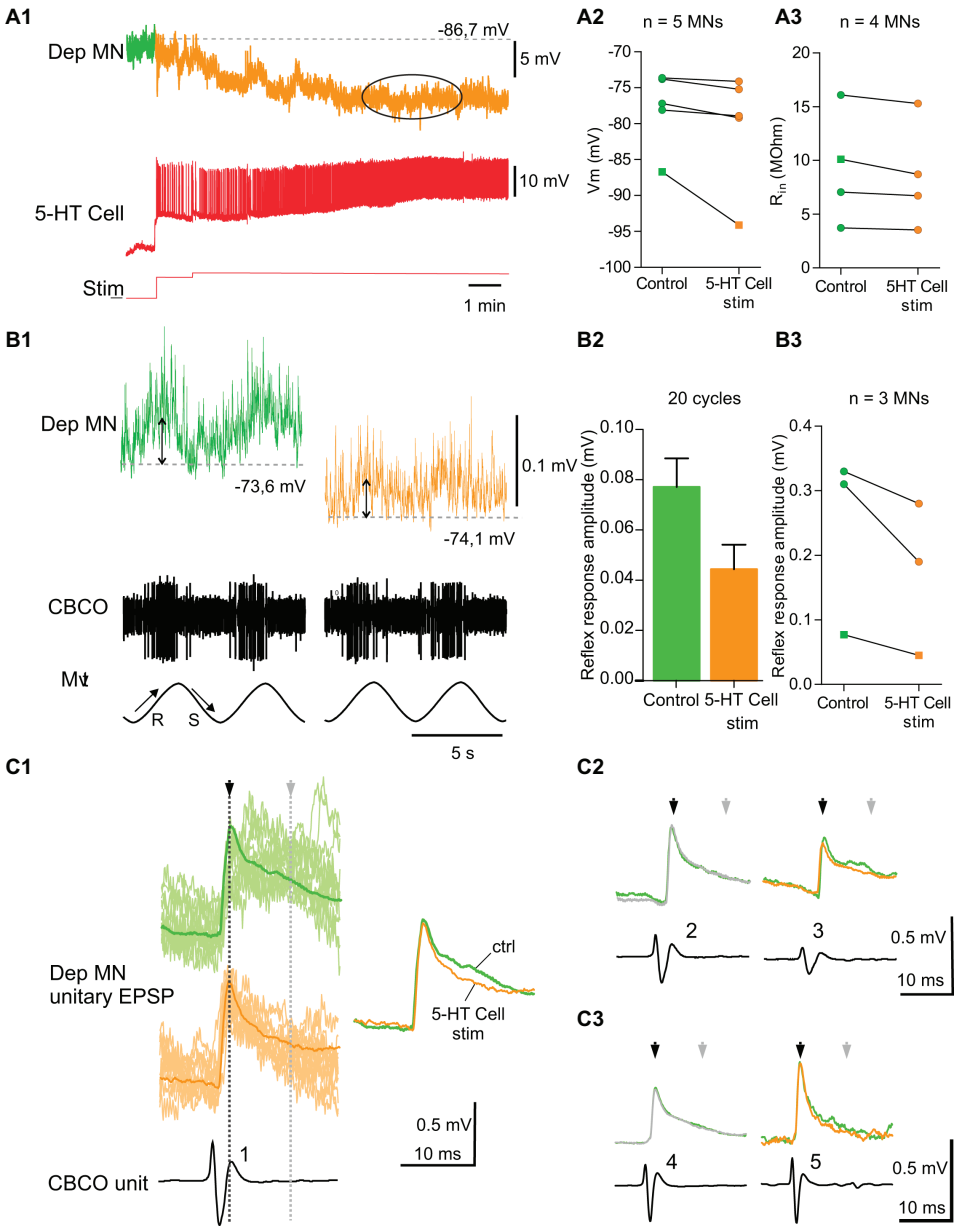
Inhibitory effects induced by 5-HT cell activation on Dep MNs. **(A1)** hyperpolarizing response of an intracellularly recorded Dep MN to the ipsilateral A1 5-HT cell intracellular stimulation. **(A2)** Inhibitory effect of 5-HT cell activation on 5 Dep MNs. Square symbol represents trace used in **A1**. **(A3)** Decrease of input resistant (*R*_in_) from four MNs when 5-HT cell are stimulated. Membrane potential of these MNs decreases also. Square symbol represents trace used in **A1**. **(B1)** Decrease of the amplitude of the sensory-motor response of an intracellularly recorded Dep MN during 5-HT cell activation (same dispositions as in Figure 4). **(B2)** In this experiment, reflex response amplitude decreases from 0.077 ± 0.011 mV (averaged from 20 cycles) to 0.045 ± 0.009 mV (averaged from 20 cycles). **(B3)** Decrease of sensory-motor response recorded from three MNs, after the 5-HT cell stimulation. Square symbol represents trace used in **A1**. **(C1)** Examples of change of unitary EPSPs’ shape induced in Dep MN that showed a hyperpolarizing response to 5-HT cell activation. In control condition and after 5-HT cell stimulation, superimposed raw data (light traces) and corresponding average EPSP traces (dark traces) are presented. **(C2,C3)** Mean EPSP amplitudes measured at peak (black arrows) and 15 ms after peak in the late part of the decay phase (gray arrows). **C2** and **C3** represent two different experiments.

Since the present work aimed at analyzing excitatory and inhibitory effects induced in intracellularly recorded Dep MNs by 5-HT cell stimulation, they are presented thereafter separately (see Figures 4, 5, respectively).

### Excitatory Effects of 5-HT Cell Activity on Depressor Motoneurons

#### Membrane Potential Depolarization

Injection of depolarizing current into the left A1 5-HT cell induced a depolarization in 10 of the 16 intracellularly recorded Dep MNs (Figure 4A). A representative recording of such a response is shown in Figure 4A1. The Dep MN response started a few seconds after the beginning of 5-HT cell stimulation, and reached a maximum depolarization of 7.8 mV after 5 min of stimulation (Figure 4A1, ellipse). After the end of 5-HT cell stimulation, the membrane potential of the Dep MN came back close to its control value within a few mins (Figure 4A1). The changes in membrane potential induced in these 10 intracellularly recorded Dep MNs after 5 min 5-HT cell stimulation are presented in Figure 4A2. The control membrane potential was averaged over 1 min before the 5-HT cell was stimulated (green part of recordings in Figure 4A2, and green dots in Figure 4A2). The maximum of the depolarizing response was measured 5 min after the beginning of 5-HT cell stimulation and averaged over 2 min (see ellipses in Figure 4A1, and blue dots in Figure 4A2). The excitatory effects of the 5-HT cell stimulation are significant on the membrane potential of recorded MNs (Figure 4A2; paired *t*-test, *p* < 0.01, *n* = 10).

#### Input Resistance Increase

Before the left A1 5-HT cell activation and 15–20 min after, the input resistance (*R*_in_) of the intracellularly recorded Dep MN was measured (Figure 4A3). In the six MNs in which *R*_in_ was measured during the depolarizing effect of 5-HT cell stimulation, an increase of *R*_in_ was observed, indicating that these depolarizations were excitatory (paired *t*-test, *p* = 0.08, *n* = 6).

#### Functional Consequence on Postural Control

The application of sinusoidal movements to the CBCO strand elicited alternately depolarizing and hyperpolarizing responses in depressor MNs (El Manira et al., 1991; Le Ray et al., 1997a,b). Release of the CBCO, which mimicked the upward movement of the leg, depolarized the depressor MNs in seven quiescent (i.e., non-rhythmically active) preparations (Figure 4B1, upward arrow; El Manira et al., 1991; Le Ray et al., 1997b). Conversely, stretch of the CBCO, which mimicked leg downward movement, hyperpolarized the depressor MNs, presumably by exciting inhibitory pathways (Figure 4B1_,_ downward arrow; Le Bon-Jego and Cattaert, 2002).

In six experiments, in which the intracellular stimulation of the ipsilateral 5-HT-cell induced an excitatory effect on the intracellularly recorded Dep MN, we also analyzed the response of this Dep MN to release movements of the CBCO. In these six Dep MNs, we observed an increase of the amplitude of the response to CBCO release. A representative example of a response induced in a Dep MN by 5-HT cell stimulation is given in Figure 4B1. In this experiment, the amplitude of the response of the Dep MN to CBCO movements (Figure 4B2) increased from 1.03 ± 0.07 mV in control conditions (green trace, double arrow) to 1.66 ± 0.96 mV after 5 min of 5-HT cell stimulation (blue trace, double arrow). When we compared the amplitude of the Dep MN responses in the six experiments, we observed a significant increase (paired *t*-test, *p* < 0.05, *n* = 6) in Dep MN response to CBCO when 5-HT cells were stimulated (Figure 4B3).

#### Coxo-Basal Chordotonal Organ -Induced Unitary EPSPs in Depressor Motoneurons

The unitary EPSPs evoked by CBCO sensory afferents were analyzed in experiments in which A1 5-HT cell stimulation induced a change of the amplitude of the response to CBCO movements. For example, in the experiment shown in Figure 4B1, the intracellularly recorded Dep MN received unitary EPSPs triggered by five different CBCO sensory units (Figure 4C1). Each unitary EPSP is shown before A1 5-HT cell stimulation (control – green trace) and after 5 min of 5-HT-cell stimulation. With each unitary EPSP, the shape of the corresponding CBCO afferent spike is presented, together with an identifying number. All the sensory units involved in the Dep MN response fired during release movement of the CBCO strand (i.e., upward movement of the leg in intact animals) and because they evoked an EPSP in a Dep MN, they were therefore involved in resistance reflex. The most remarkable result of this analysis was that the amplitude of unitary EPSPs was only slightly affected if at all after 5-HT-cell stimulation. For one unit, the peak amplitude of the unitary EPSP (Figure 4C1, black arrowhead) was slightly increased (unit 1). One out of the five unitary EPSP was unchanged (unit 2). The three other unitary EPSPs were even reduced (units 3, 4, and 5 – see black arrow heads). This result was very surprising because in the presence of 5-HT, the input resistance of this recorded Dep MN was increased by 32%, leading us to expect a proportional increase in the amplitude of individual EPSPs. Indeed, as was shown for 5-HT application in Cattaert et al. (2010), the increase of the amplitude of the MN response to CBCO movements was mainly associated with late part of EPSP increase and suggests that the effect is indirect and mediated by polysynaptic sensory-motor pathways (polysynaptic component of unitary EPSPs – see also overdraws in Figure 4C1, showing more polysynaptic events in blue traces). Such a marked increase in the late part of EPSP was also observed in the experiment reported in Figure 4C1 (gray arrowhead). Indeed four units presented a marked increase in the time constant of the repolarizing phase of the EPSP (units 1, 3, 4, and 5). Previously, we demonstrated that such a prolonged EPSP depolarization was responsible for the increase in the amplitude of MN response to CBCO movements in preparations exposed to applied 5-HT due to increased summation of EPSPs (Le Bon-Jego et al., 2004).

### Inhibitory Effects of 5-HT Cell Activity on Depressor Motoneurons

#### Membrane Potential Hyperpolarization

Injection of depolarizing current into the left A1 5-HT cell induced a hyperpolarization in five of the 16 intracellularly recorded Dep MNs (Figure 5A). A representative recording of such a response is shown in Figure 5A1. The Dep MN response started a few seconds after the beginning of 5-HT cell stimulation, and reached a maximum hyperpolarization of −7.4 mV after 5 min of stimulation (Figure 5A1, ellipse). The results of five hyperpolarizing responses are presented in Figure 5A2. The control membrane potential was averaged over 1 min before the 5-HT cell was stimulated (see green part of recordings in Figure 5A1, and green dots in Figure 5A2). The maximum of the hyperpolarizing response was measured 5 min after the beginning of 5-HT cell stimulation and averaged over 2 min (see ellipses in Figure 5A1, and orange dots in Figure 5A2). A slight hyperpolarization was observed (Figure 5A2, paired *t*-test, *p* = 0.06, *n* = 5) in all five MNs.

#### Input Resistance Decrease

Before the left A1 5-HT cell activation and 15–20 min after, the input resistance (*R*_in_) of the intracellularly recorded Dep MN was measured (Figure 5A3). In the four MNs in which *R*_in_ was measured during the hyperpolarizing effect of 5-HT cell stimulation, a significant decrease of *R*_in_, was observed (paired *t*-test, *p* < 0.05, *n* = 4).

#### Functional Consequence on Postural Control

In three experiments, in which the intracellular stimulation of the ipsilateral 5-HT cell induced an inhibitory effect on the intracellularly recorded Dep MN, we also analyzed the response of this Dep MN to release movements of the CBCO. In these three Dep MNs, we observed a decrease of the amplitude of the response to CBCO release. A representative example of a hyperpolarizing response induced in a Dep MN by 5-HT cell stimulation is given in Figure 5B1. In this experiment, the amplitude of the response to CBCO movements measured in the Dep MN (Figure 5B2) decreased from 0.077 ± 0.011 mV in control condition (green trace) to 0.045 ± 0.009 mV after 5 min of 5-HT cell stimulation (orange trace). This effect was consistent in the three Dep MNs that were recorded in three different preparations (Figure 5B3, paired *t*-test, *p* = 0.06, *n* = 3).

In the experiments, in which 5-HT cell stimulation induced a decrease of the amplitude of the MN response to CBCO movements, the analysis of unitary EPSPs showed that very few changes occurred in EPSP shapes (Figure 5C1). The only noticeable modification was a decrease in the late decay phase (Figure 5C1, gray arrow). In this figure, the unitary EPSPs identified in two Dep MNs are presented (Figures 5C2,C3). In these two experiments, the faster decay time induced after 5-HT cell stimulation was likely due to inhibition of the polysynaptic pathways (as indicated by the more regular exponential decay of the EPSPs in Figure 5C, orange traces – see also overdraws in Figure 5C1, showing fewer polysynaptic events in orange traces). Concomitantly, in these experiments, the stimulation of the 5-HT cells also induced a drop in input resistance of the Dep MNs (Figure 5B3). This result is in accordance with the fact that the decay time constant was faster in some EPSPs (for example, units 1, 3, and 5 in Figure 5C1).

### The Effect of 5-HT Cell Stimulation on the Depressor Motoneurons Is Direct

**Figure 6:**
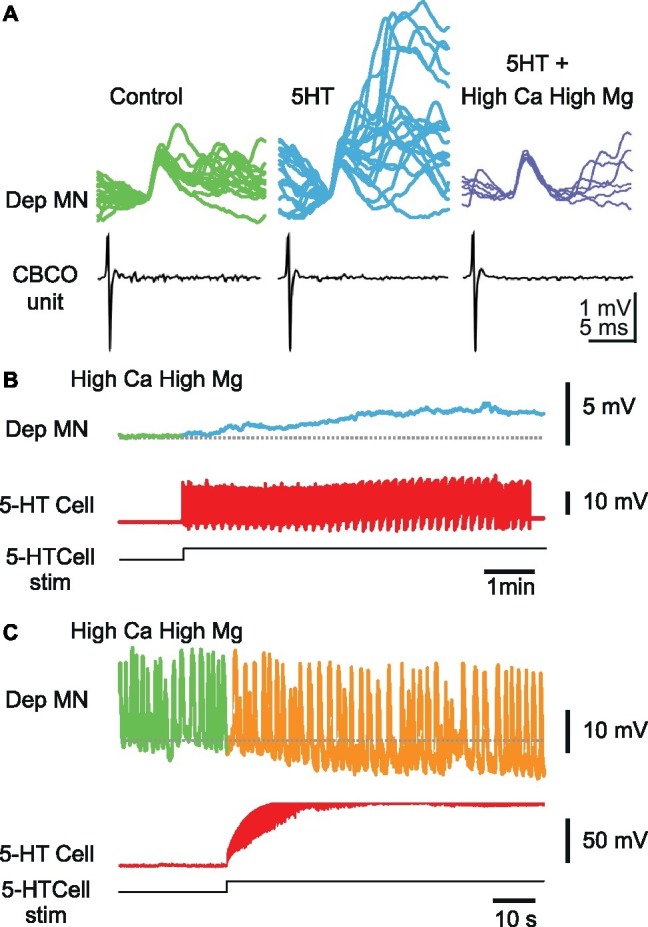
Effect of 5-HT cell stimulation on Dep MNs persists in high-divalent cation solution. **(A)** High-divalent cation solution raises the threshold for spikes. The effect of high-divalent cation solution was tested on unitary EPSPs recorded from a Dep MN. In control situation, the CBCO unit (black trace) evoked an EPSP in the intracellularly recorded Dep MN (green trace) in which very few polysynaptic events occurred. After 10 min of perfusion of 5-HT (10 μM) in the bathing medium, the polysynaptic pathways were activated (see the numerous and variable events present in the decay phase of the monosynaptic EPSP). If the 5-HT was applied while the preparation was perfused with high-divalent cation solution, no polysynaptic event occurred (dark blue traces). **(B)** Persistence of a depolarizing response induced by 5-HT cell stimulation in high-divalent cation solution. **(C)** Persistence of a hyperpolarizing response induced by 5-HT cell stimulation in high-divalent cation solution. Note that this Dep MN was producing pacemaker properties, the frequency of which decreased during 5-HT cell stimulation.

We wanted to determine if a part of the effect of A1 5-HT cell stimulation onto depressor MNs could be direct. To test for this possibility, we used high-divalent cation solution (2.5 × Ca^2+^, 2.5 × Mg^2+^) was used to raise the threshold for triggering a spike, and thereby minimizing the polysynaptic pathways. The efficacy of this procedure was assessed by the fact that sensory-motor pathways from CBCO units to Dep MNs were drastically eliminated (Figure 6A). In this example, in control condition (Figure 6A, left), the CBCO unit (black trace) triggered an EPSP with very few polysynaptic events (green traces). The perfusion of 10 μM 5-HT in the bathing medium (Figure 6A, middle) recruited these polysynaptic pathways. However, high-divalent cation solution prevented this recruitment (Figure 6A, right).

In the presence of high-divalent cation solution (perfusion restricted onto the two last thoracic ganglia), the activation of the A1 5-HT-cell could still induce changes in membrane potential of the intracellularly recorded Dep MN, either depolarizing (Figure 6B), or hyperpolarizing (Figure 6C), suggesting that the effects of ipsilateral A1 5-HT cell involved monosynaptic connections from 5-HT cell onto Dep MNs. Note that the Dep MN presented in Figure 6C kept a rhythmic activity in the presence of high-divalent cations solution. Plateaus (>20 mV) were produced all along the recording (visible oscillations represent mainly the plateaus). Despite this exceptional activity, 5-HT cell was nevertheless capable of hyperpolarizing Dep membrane potential.

### Monotonal Distribution of Effects of 5-HT Cell Activity on Depressor Motoneurons

Because local micro-ejection of 5-HT produced inhibitory or excitatory effects depending on the site of ejection (respectively on the initial segment or in the center of the neuropile) (Bacqué-Cazenave et al., 2013), one of the aims of this study was to test if 5-HT cell stimulation could also evoke excitatory and inhibitory effects in Dep MNs. The data presented above indicate that this is the case. It seems therefore likely that abdominal 5-HT cells participate in both types of modulation of Dep MN activity.

However, this result raised the question of the significance of these opposed effects. Indeed, this result may be surprising because all animals included in the present study were isolated animals, and we would have expected mostly excitatory effects. It could be that two types of responses correspond to two types of MN pools – one involved in subordinate animal responses and the other involved in dominant animal responses as shown by Cattaert et al. (2010). To test if the two types of responses correspond to two types of MN pools, we analyzed the distribution of the effects induced by 5-HT cell stimulation on Dep MNs.

**Figure 7:**
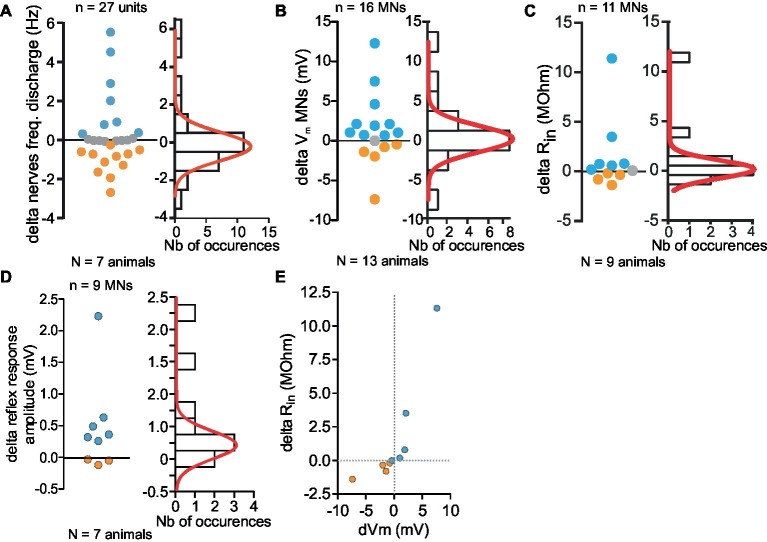
Monotonal distribution of effects of 5-HT cell activity on Depressor MNs. **(A–D)** Variety of responses magnitude (firing frequency, **A**; *V*_m_, **B**; *R*_in_, **C**; reflex response, **D**) measured in *in vitro* preparations (left panel). Distribution of all changes and their distribution histogram fitted with a Gaussian curve (right panel). **(E)** Correlation between the changes of input resistance (*R*_in_) and membrane potential changes of eight MNs. Orange and blue dots represent respectively inhibitory and excitatory effects induced by stimulation of 5-HT cell. All depolarizing changes of Dep MNs were associated with an increase of *R*_in._ Hyperpolarizing changes were associated with a *R*_in_ decrease.

The distribution of the changes in frequency observed in the various MNs (*n* = 27) recorded in motor nerves over all experiments (*N* = 7) was fitted with a Gaussian distribution curve (Figure 7A), indicating that both excitatory and inhibitory responses could obey a single distributing law. Indeed the change in frequency of Dep MN ranged from −2.69 to +5.52 Hz, but no clear separation between two groups was revealed.

A similar conclusion was drawn from the effects on membrane potential (Figure 7B), *R*_in_ (Figure 7C) and sensory-motor response change (Figure 7D). There is a clear correlation between the change in *R*_in_ and the amplitude of the depolarization suggesting that intrinsic and network effects are parallel consequences of 5-HT cell stimulations (Figure 7E).

Note that the overall response on the leg postural network is excitatory because the number of measurements in favor of excitatory effects is larger and their values are also larger than for inhibitory effects. Although the various effects on discharge frequency, membrane potential, and input resistance were variable among the MN population (excitatory, inhibitory, and no change), it is interesting to note that functionally, the amplitude of the response to CBCO movement increases from 1.04 ± 0.29 mV in control to 1.68 ± 0.49 mV after 5-HT cell stimulation (paired *t*-test, *p* < 0.05, *n* = 9, *N* = 7).

### 5-HT Cell Stimulation Enhances the Response to 5-HT Motoneurons to Abdominal Sensory Cues

**Figure 8:**
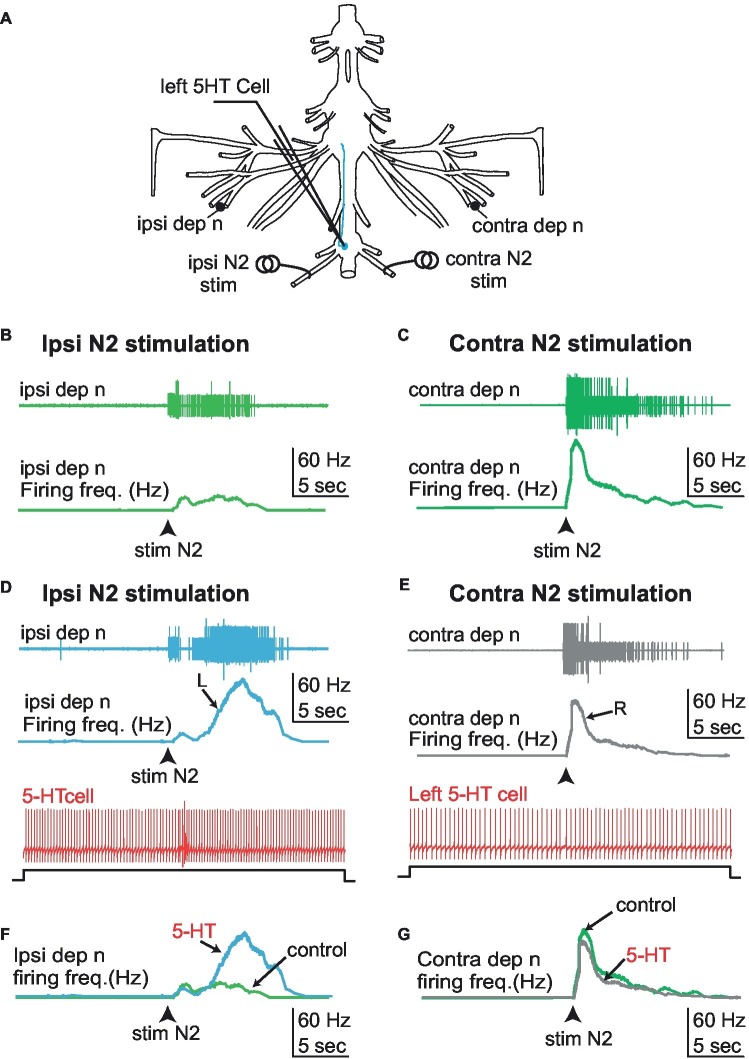
Effect of 5-HT cell stimulation on the response pattern of the depressor nerve to N2 sensory stimulation. **(A)** Experimental disposition. **(B)** Stimulation of the left (ipsilateral) N2 sensory nerve while keeping the left 5-HT cell silent activates the left depressor nerve (light green). **(C)** Similarly, stimulation of the right (controlateral) sensory nerve while keeping the left 5-HT cell silent activates the right depressor nerve (dark green). **(D)** Driving the left 5-HT cell by current injection enhances the response of the left (ipsilateral) depressor nerve (blue trace) to stimulation of the left (ipsilateral) N2 sensory nerve. **(E)** By contrast, the same activation of the left 5-HT cell during right (contralateral) N2 sensory nerve stimulation does not enhance the response of the right (contralateral) depressor nerve (gray trace). **(F,G)** Comparison of the frequency response of the ipsi/contra depressor nerves to left N2 sensory nerve stimulation in the absence and presence of 5-HT cell stimulation. Whereas a noticeable increase of the response is observed on side ipsilateral to 5-HT cell stimulation **(F)**, no effect is observed in the contralateral side **(G)**. R, right; L, left.

We have shown that left 5-HT cells from the first abdominal ganglion modulate the activity of MNs from ipsilateral fifth ganglion and their responses to proprioceptive inputs from the same leg joint. Here we address the question of the effect of the same 5-HT cell onto sensory inputs from other parts of the body. More specifically, we measured the response of the depressor nerve units to stimulation of the N2 sensory nerve of the first abdominal ganglion (that contains the axons of fringe hair afferents), with and without activation of the left 5-HT neuron (Figure 8, *N* = 5). As a control, we measured the response of the depressor nerve to ipsilateral N2 sensory stimulation while the 5-HT neuron was kept silent by injecting negative DC current in the cell soma (Figures 8B,C). We repeated the test as the 5-HT neuron was driven at a rate of ~10 Hz by injecting positive current. We found that the response of the depressor nerve to N2 sensory stimulation was enhanced when the 5-HT neuron was active (Figures 8D,F) as compared to when it was silent (Figure 8B). Conversely, 5-HT neuron stimulation did not change the response of the depressor nerve to stimulation of the contralateral N2 sensory stimulation (Figures 8C,G,E). The lack of inhibitory effect of 5-HT cell stimulation on Dep MNs’ activity in these experiments may be surprising. This is likely due to the absence of spontaneous activity of Dep MNs, which probably resulted from the long-lasting period (>1.5 h) of the preparation before starting recordings. This result generalizes the modulatory role of A1 5-HT neurons onto fifth leg postural network, and confirms that this modulatory effect is limited to the ipsilateral side.

## Discussion

In a previous paper (Bacqué-Cazenave et al., 2013), it was shown that 5-HT could elicit excitatory or inhibitory effects, depending on where it was locally applied. When micro-ejections were performed close to the initial segment where 5-HT immunoreactive fiber form boutons onto MN axon, the induced effect was inhibitory, while when 5-HT was micro-ejected in the center of the neuropile where no direct contact between 5-HT immunoreactive fibers and MN dendrite exist, an excitatory effect was observed. Here, in postural networks of isolated animals, we show that (1) the activation of a single 5-HT cell can induce mixed excitatory and inhibitory effects on walking leg MNs; (2) these effects induce functional changes in the walking leg postural network by modifying the amplitude of the response of MNs to mechanosensory inputs; (3) the induced effects are multiple and cooperative, and they involve modifications of the intrinsic properties (input resistance, membrane potential) of Dep MNs – as shown in Le Bon-Jego et al. (2004) when 5-HT was bath applied; and (4) the effects on intrinsic properties of Dep MNs are direct, while the effects on sensory-motor responses also involve polysynaptic pathways.

### 5-HT Cell Stimulation Has a Variety of Effects on the Leg Postural Network

Excitatory responses induced in Dep MNs by A1 5-HT cell stimulation consist of: (1) a tonic depolarization directly linked to the increase of input resistance and (2) an increase of the amplitude of MN response to CBCO movements linked to the prolonged repolarizing phase of EPSPs (likely due to recruitment of polysynaptic sensory-motor pathways). All these features were observed after bath application of 5-HT (10 μM) in some communal animals (Le Bon-Jego et al., 2004) and in dominant animals (Cattaert et al., 2010) or by local pressure micro-ejection in the central part of the neuropile in isolated animals (Bacqué-Cazenave et al., 2013) By contrast, inhibitory responses induced in Dep MNs by A1 5-HT cell stimulation consist of: (1) a tonic hyperpolarization associated with a decrease of input resistance and (2) a decrease of the amplitude of MN response to CBCO movements linked to a faster repolarizing phase of EPSPs (likely due to the failure to recruit polysynaptic sensory-motor pathways). All these features were observed after bath application of 5-HT (10 μM) in some communal animals (Le Bon-Jego et al., 2004) and in subordinate animals (Cattaert et al., 2010) or by local pressure micro-ejection close to the initial segment of Dep MNs in isolated animals (Bacqué-Cazenave et al., 2013). These results indicate that the activity of a single A1 5-HT cell is capable of inducing each of these cooperative effects (either excitatory or inhibitory) on the leg postural circuit.

Using local micro-application of 5-HT into the fifth thoracic ganglion that contains the postural network, it was shown that these two opposed responses to 5-HT involve different regions of MN architecture. While inhibitory effects from 5-HTcells may involve classic synaptic contacts close to the initial segment of Dep MNs, more central excitatory effects on the dendritic arbor are most likely due to paracrine mechanisms (Bacqué-Cazenave et al., 2013). The present results indicate that a single 5-HT cell can trigger both types of 5-HT release: inhibitory synaptic action onto the initial segment and excitatory paracrine mechanisms on dendritic arbor. This finding raises several questions: (1) Why do some MNs respond to 5-HT cell stimulation with a paracrine excitatory effect while other MNs respond with a synaptic inhibitory effect? (2) How does the motor network process or condition these opposed responses? (3) Are these opposed effects related to the heterogeneity of MNs in a given pool? and (4) What could be the functional schema in which 5-HT cells control network activity?

### 5-HT Cell Induces Both Synaptic and Paracrine Mechanisms

Our results indicate that excitatory and inhibitory effects observed in MNs after 5-HT cell stimulation are in conformity with the effects of local micro-application of 5-HT on dendritic arbor and initial segment, respectively (Bacqué-Cazenave et al., 2013). Moreover, those effects persist in high-divalent cation Ringer, indicating a direct effect of 5-HT cells’ axonal branches on the two types of receptor sites onto MNs.

5-HT action is likely not due to a spillover of neurotransmitter from a synaptic site because this would have induced inhibition before paracrine activation of excitatory sites could occur, which was not observed. Moreover, in such a scenario, it is likely that the inhibitory effect would have blocked any subsequent excitatory response due to mutual interactions between the two molecular pathways as was shown in the lateral giant synapse of crayfish (Lee et al., 2008). In addition, we do not know how much time it would take for the 5-HT spillover to reach the dendritic paracrine receptors. In their report, Lee et al. (2008) observe the reversal of the response from inhibitory to excitatory during the wash, after at least 15 min for short applications of high concentration (50–1,000 μM) of 5-HT.

This consideration implies that some parts of the axonal arbor release 5-HT in a paracrine way to activate 5-HT receptors on MN dendrites to elicit an excitatory response, while other parts of the axonal arbor of the same 5-HT cell are involved in synapses at the initial segment of MNs triggering inhibitory responses (see Figure 10 in Bacqué-Cazenave et al., 2013).

### Excitatory vs. Inhibitory Effects of 5-HT Cell Stimulation Onto Motoneurons

5-HT cell stimulation induces excitatory response in some MNs and inhibitory responses in other MNs, and these responses are not segregated into two separated pools but rather obey a single Gaussian-like distribution (Figure 7). This finding could indicate that the two types of effects occur simultaneously in different mixtures for the different MNs. However, this situation, in which both excitatory and inhibitory pathways are activated, seems unlikely because these two pathways are competitive and eventually one would overcome the other (Lee et al., 2008).

Another interpretation may be suggested in which the MNs “decide” the type of response they will make to 5-HT cell stimulation. When the stimulation was made several times, the same MN would respond in the same way each time, and this response would always be either excitatory or inhibitory but not mixed. So we may conclude that, by an unknown mechanism, each MN will be controlled either by paracrine excitatory process or by synaptic inhibitory synapse. In addition, the intensity of the response is also specific for each MN, being large, small, or absent. Altogether, those settings allow a Gaussian-like distribution of responses from negative to positive. This distribution is reminiscent of a homeostatic mechanism that would set 5-HT receptors (internalization, phosphorylation, etc.) of each MN depending on its activity in order to adjust its response to 5-HT.

### Network Effect on 5-HT Neuromodulation of Motoneuron Activity

In the present report, we showed that MN responses to 5-HT cell stimulation persisted in high-divalent cation solution. This condition eliminates polysynaptic pathways by raising spike threshold. However, in the physiological situation, the activation of a MN pool would result in the inhibition of the antagonistic MN pool (Pearlstein et al., 1998). Thereby, secondary inhibitions can be observed.

### Functional Consequences of Mixed Excitatory/Inhibitory Effects Induced by 5-HT Cell Activity

The above discussion pointed out a possible homeostatic mechanism allowing 5-HT cells to adapt their level of control on MNs depending on each MN’s activity. Active MNs will tend to express 5-HT receptors leading to inhibition, whereas resting MNs will favor 5-HT receptors leading to excitation. If this mechanism exists, its time course must be slow, based on the level of 5-HT and on the activity of MNs. We propose a mechanism similar to the one described during social status formation concerning future subordinate crayfish (Yeh et al., 1996).

During social status formation in isolated animals, 5-HT application elicits an excitatory effect on lateral giant fiber and on its mechanosensory synaptic inputs. This effect is gradually decreased and replaced by an inhibitory effect in the animal that loses fights and becomes submissive (Yeh et al., 1997). In the present report, 3 weeks of social isolation prior to experiments erased the animals’ previous social status. The observed diversity of 5-HT effects was therefore not related to their social status. Moreover, excitatory and inhibitory effects were observed in the same preparation. Nevertheless, it is possible to invoke similar mechanisms controlling the effects of 5-HT onto MNs and depending on their level of activity and depending on the level of 5-HT rather than on social status.

## Concluding Remarks

Since the firing rate of a first abdominal 5-HT cell seems to control each ipsilateral fifth leg MN either with excitatory or inhibitory effects that obey a single Gaussian distribution, we assume that the present results point to a homeostatic mechanism relying on each MN’s activity. In addition, since the firing of the 5-HT cell is driven notably by mechanosensory inputs occurring during tail-flip (Issa et al., 2012), it may represent the result of integration of various cues about behavioral environment that precisely tunes 5-HT excitatory and inhibitory effects on each MN to produce a double time-scale control of their individual activities: (1) rapid modulation of each MN activity is controlled by the firing rate of 5-HT cells but (2) the sign and magnitude of these rapid effects would be tuned in a much slower way.

## Data Availability Statement

The datasets generated for this study are available on request to the corresponding author.

## Author Contributions

JB-C made the experiments, data analysis, writing, and editing the manuscript. PF and JD helped in writing and editing the manuscript. FI made the experiments, data analysis, writing, and editing the manuscript. DE contributed in designing Experiments, writing, and editing the manuscript. DC helped in designing experiments and made the experiments, data analysis, writing, and editing the manuscript.

### Conflict of Interest

The authors declare that the research was conducted in the absence of any commercial or financial relationships that could be construed as a potential conflict of interest.
